# Equilíbrio Dinâmico e Mobilidade Explicam a Qualidade de Vida na ICFEP, Superando Todos os Outros Componentes da Aptidão Física

**DOI:** 10.36660/abc.20190080

**Published:** 2020-05-12

**Authors:** Cristine Schmidt, Mário Santos, Lucimere Bohn, Bruno Miguel Delgado, Daniel Moreira-Gonçalves, Adelino Leite-Moreira, José Oliveira

**Affiliations:** 1 Faculdade de Desporto Universidade do Porto Centro de Investigação em Actividade Física, Saúde e Lazer Porto Portugal Faculdade de Desporto da Universidade do Porto (FADEUP), Centro de Investigação em Actividade Física, Saúde e Lazer (CIAFEL), Porto – Portugal; 2 Instituto de Ciências Biomédicas Abel Salazar Universidade do Porto Unidade de Pesquisa Multidisciplinar em Biomedicina Porto Portugal Instituto de Ciências Biomédicas Abel Salazar (ICBAS), Universidade do Porto, Unidade de Pesquisa Multidisciplinar em Biomedicina, Porto – Portugal; 3 Centro Hospitalar do Porto Departamento de Cardiologia Porto Portugal Centro Hospitalar do Porto, Departamento de Cardiologia, Porto – Portugal; 4 Centro Hospitalar do Porto Porto Portugal Centro Hospitalar do Porto, Porto – Portugal; 5 Faculdade de Medicina Universidade do Porto Unidade de Investigação Cardiovascular Porto Portugal Faculdade de Medicina da Universidade do Porto (FMUP), Unidade de Investigação Cardiovascular (UniC), Porto - Portugal

**Keywords:** Insuficiência Cardíaca/fisiopatologia, Equilíbrio Trabalho-Vida/métodos, Aptidão Física, Qualidade de Vida, Exercícios Respiratórios, Composição Corporal

## Abstract

**Fundamento:**

A aptidão física é um importante determinante da qualidade de vida (QV) em pacientes com insuficiência cardíaca com fração de ejeção preservada (ICFEP). No entanto, ainda não se sabe como os diferentes componentes da aptidão física se relacionam com as dimensões específicas da QV em pacientes com ICFEP.

**Objetivo:**

Avaliar a associação entre diferentes componentes da aptidão física e dimensões da QV em pacientes com ICFEP, e examinar quais componentes da aptidão física foram independentemente associados à QV.

**Métodos:**

Os pacientes com ICFEP foram avaliados quanto à aptidão física [equilíbrio dinâmico e mobilidade (“teste *8-feet Up-and-go* ”), força da parte superior do corpo (Teste de força de preensão manual), aptidão cardiorrespiratória (ACR) (teste de caminhada de 6 minutos) e composição corporal (índice de massa corporal)] e para QV ( *Minnesota Living With Heart Failure Questionnaire* ). Uma correlação parcial foi utilizada para verificar a associação entre os componentes da aptidão física e as dimensões da QV. A análise das dimensões dos preditores independentes de QV foi realizada através da análise de regressão linear multivariada *stepwise* . A significância estatística foi estabelecida em p <0,05.

**Resultados:**

Tanto a ACR quanto o equilíbrio dinâmico e a mobilidade estão significativamente associados ao escore total e às dimensões físicas da QV (p <0,05), mas apenas o equilíbrio dinâmico e a mobilidade foram concomitantemente associados à dimensão emocional (r = 0,597; p = 0,004). O equilíbrio dinâmico e a mobilidade foram associados de forma independente ao escore total (β = 0,651; r^2^ = 0,424; p = 0,001), e as dimensões física (β = 0,570; r^2^ = 0,324; p = 0,04) e emocional (β = 0,611; r^2^ = 0,337 p = 0,002) da QV.

**Conclusão:**

Nossos dados sugerem que o equilíbrio dinâmico e a mobilidade avaliam melhor a QV do que a ACR, comumente medida na prática clínica. Ainda não se sabe se as intervenções direcionadas especificamente ao equilíbrio dinâmico e à mobilidade têm diferentes impactos na QV. (Arq Bras Cardiol. 2020; 114(4):701-707)

## Introdução

A insuficiência cardíaca com fração de ejeção preservada (ICFEP) é responsável por metade de todos os casos de IC na população do mundo desenvolvido.^1^ A manifestação mais comum da doença é a intolerância ao exercício, que afeta a capacidade do paciente de lidar com as atividades da vida diária e reduz sua qualidade de vida (QV).^2^ Além disso, a QV está relacionada a desfechos ruins, como maior frequência de readmissão hospitalar e maiores taxas de mortalidade.^3^ Apesar de sua alta prevalência e prognóstico ruim, a ICFEP permanece uma doença sem tratamento aprovado que melhore a sobrevida.^4^ Portanto, as recomendações atuais para o tratamento desses pacientes destacam a importância de se concentrar em tratamentos eficazes, capazes de aliviar os sintomas e melhorar significativamente a QV.^5^

Níveis reduzidos de aptidão física estão associados à baixa QV em pacientes com ICFEP.^6 , 7^ É importante ressaltar que o treinamento com exercícios mostrou melhora na aptidão física, juntamente com uma melhora nos sintomas e na QV.^6 , 7^ Como a aptidão física e a QV estão relacionadas entre si, concentrar-se na aptidão física com programas de exercícios físicos pode ser uma estratégia eficaz para atingir as recomendações para o manejo de pacientes com ICFEP.^5^ Entretanto, a aptidão física é um construto de múltiplos componentes (por exemplo, equilíbrio dinâmico e mobilidade, aptidão muscular, aptidão cardiorrespiratória (ACR) e composição corporal)^8^ e, em paralelo, a QV é um construto multidimensional (por exemplo, dimensão geral, emocional e física).^9^ Até agora, ainda não se sabe como os diferentes componentes da aptidão física se relacionam com as dimensões específicas da QV em pacientes com ICFEP. Até o momento, foi demonstrado apenas que uma maior ACR está associada a uma melhor QV, principalmente em relação à dimensão física.^7 , 10^ Entretanto, a influência de outros componentes da aptidão física nas dimensões da QV é relativamente desconhecida. Portanto, o esclarecimento dessa questão pode ter implicações clínicas importantes no desenho de programas intervencionistas específicos para pacientes com ICFEP, visando o componente de aptidão física, o qual têm o maior impacto na QV em geral ou em uma de suas dimensões reduzidas.

Portanto, os objetivos do presente estudo são duplos: i) avaliar a associação entre diferentes componentes da aptidão física (ACR, força da parte superior do corpo, equilíbrio dinâmico e mobilidade e composição corporal) e as dimensões da QV (total, física e emocional) em pacientes com ICFEP, e ii) examinar quais componentes da aptidão física estão independentemente associados a diferentes dimensões da QV nessa população específica.

## Métodos

### Desenho do estudo

Trata-se de um estudo transversal realizado em um hospital público português (Centro Hospitalar do Porto - Hospital de Santo Antônio, Porto) com uma amostra de conveniência de ICFEP. Os critérios de inclusão foram: diagnóstico de ICFEP de acordo com as diretrizes da Sociedade Europeia de Cardiologia.^11^ Os pacientes foram excluídos se apresentassem angina instável, síndrome coronariana aguda como diagnóstico primário, estenose aórtica grave sintomática, embolia pulmonar aguda, miocardite aguda, insuficiência cardíaca descompensada, hipertensão não-controlada, arritmias ventriculares complexas, disfunção renal grave, doença pulmonar obstrutiva crônica grave, condições médicas ou ortopédicas que impediam a deambulação independente e a realização do teste ergométrico.

Os pacientes potencialmente elegíveis para participar do estudo foram identificados nos prontuários clínicos do departamento de cardiologia do hospital. Um total de 30 pacientes foram convidados por telefone por um cardiologista. Desses, 24 pacientes (17 mulheres e 7 homens) aceitaram participar do estudo. O estudo foi aprovado pelo Comitê de Ética do Centro Hospitalar do Porto - Hospital de Santo Antônio (N / S: 2015.125) e atendeu aos padrões éticos da Declaração de Helsinque.

Os dados foram coletados de novembro de 2016 a setembro de 2017 em um único dia no hospital.

### Coleta de dados

#### Pressão sanguínea

Um pesquisador treinado realizou medições da pressão arterial (PA) após 10 minutos em repouso na posição sentada. A PA foi avaliada (Colin, BP 8 800; Critikron, Inc., EUA) em ambos os braços, e o braço com a PA mais alta foi utilizado. PA sistólica e PA diastólica foram calculadas como a média de 3 leituras, com 2 minutos de intervalo entre elas. Leituras adicionais foram realizadas quando as diferenças entre as leituras excederam 5 mmHg.^12^

## Coleta de sangue e testes bioquímicos

O sangue venoso periférico (15 mL) foi coletado em um tubo com EDTA. Os tubos com EDTA foram imediatamente colocados no gelo e deixado para coagular por 30 minutos antes da centrifugação por 15 minutos a 1000xg. O plasma foi dividido em alíquotas e armazenado a -80ºC para análise bioquímica. O peptídeo natriurético cerebral (BNP) foi quantificado em um laboratório certificado utilizando o imunoensaio de micropartículas por quimiluminescência (ARCHITECT BNP).

## Medidas antropométricas e de composição corporal

A altura corporal (cm) foi medida com o indivíduo na posição ereta, utilizando um estadiômetro (Holtain Ltd., Crymmych, Reino Unido).^13^ O peso (kg), índice de massa corporal (IMC; kg · m^2^), massa gorda (%) e massa gorda livre (kg)) foram medidos com pacientes vestindo roupas leves, utilizando um analisador eletrônico de composição corporal segmentar (Tanita, BC-418, Tóquio, Japão). A massa gorda e a massa gorda livre foram medidas utilizando impedância bioelétrica. Foi pedido aos pacientes que jejuassem por 10 a 12 horas, evitassem exercícios físicos vigorosos e ingestão de álcool antes de serem submetidos às mensurações. A circunferência da cintura (cm) foi mensurada no ponto médio entre a última costela e a crista ilíaca, no final de uma expiração normal.^14^ A obesidade foi determinada como IMC igual ou superior a 30 kg/m^2^.^15^

## Classificação funcional

Os pacientes foram classificados pelo médico em subgrupos com base em seus sintomas utilizando a classe funcional da *New York Heart Association* (NYHA). Os sintomas dos pacientes baseiam-se em quanto são limitados durante a atividade física (classe I a IV).^16^

## Avaliação Ecocardiográfica

O ecocardiograma transtorácico na posição supina foi realizado utilizando um aparelho de ultrassonografia cardiovascular modelo Vivid E95^®^ (GE Healthcare). Todas as medidas ecocardiográficas quantitativas foram realizadas por um único avaliador, cego para os resultados das outras avaliações, utilizando uma estação computadorizada de análise *off-line* . O pico da velocidade tecidual diastólica precoce foi medida no anel mitral septal e lateral. A velocidade do influxo mitral foi avaliada por Doppler de onda pulsada no corte apical de 4 câmaras, posicionando o volume da amostra na extremidade dos folhetos mitrais. A razão E /e’ foi calculada como a onda E dividida pelas velocidades de e’. A massa do ventrículo esquerdo (VE) foi estimada a partir das dimensões lineares do VE e indexadas à área da superfície corporal, conforme recomendado pelas diretrizes da ESC.^17^ A hipertrofia do VE foi definida como a massa do VE indexada à área da superfície corporal (índice de massa do VE) >115 g/m^2^ em homens ou >95 g/m^2^ em mulheres. Os volumes do VE foram estimados utilizando-se o método de Simpson modificado, usando os cortes apicais de 4 e 2 câmaras, e a fração de ejeção do VE foi derivada dos volumes da maneira padrão. O volume do átrio esquerdo (AE) foi estimado pelo método dos discos, utilizando os cortes apicais de 4 e 2 câmaras em um quadro de pressão sistólica final anterior à abertura da válvula mitral e foi indexado à área da superfície corporal para derivar o índice de volume do AE.

## Aptidão física

**Equilíbrio e mobilidade dinâmicos** . Foram avaliados através do teste *8-foot up and go* (8FUG).^18^ O paciente inicia a avaliação na posição sentada. Após um sinal, o paciente deve se levantar, andar 2,44 m (o equivalente a aproximadamente 2,5 m), dar a volta ao redor de um cone e retornar à posição inicial o mais rápido possível.^18^ Os pacientes fizeram duas tentativas de realizar o teste. O tempo (em segundos) para concluir cada tentativa foi medido com um cronômetro e o resultado considerado foi a tentativa com o tempo mais curto.^19^

**Força da parte superior do corpo** . A força de preensão manual (kg) foi medida isometricamente utilizando um dinamômetro *Lafayette Instrument Hand* (Modelo 78010, 78011, Indiana, EUA). Ambos os braços foram medidos três vezes com os pacientes na posição sentada, com o ombro aduzido e rotação neutra, o cotovelo flexionado a 90° e o antebraço e punho em posição neutra. A média entre as tentativas foi utilizada como o escore final para cada braço.^19^

**Aptidão cardiorrespiratória com avaliação das trocas gasosas pulmonares** . Foi avaliada através do teste de caminhada de 6 minutos (TC6M), em um corredor desobstruído com 25 m de comprimento. Os participantes foram instruídos a percorrer a distância máxima em 6 minutos. Paradas para descanso eram permitidas quando os pacientes consideravam necessário. O TC6M foi realizado com um analisador de gases portátil (K4b2, Cosmed, Roma, Itália) e um monitor de frequência cardíaca (Polar Electro Oy, Kempele, Finlândia). A captação de oxigênio (VO_2_; mL/min-1/kg-1) e a frequência cardíaca (FC; bpm) foram medidas direta e continuamente. As medidas respiratórias e de FC foram coletadas respiração-a-respiração e batimento-a-batimento, respectivamente, e, em seguida, os dados foram calculados para obter-se a média com intervalos de 5 s. Os dados foram calculados como a média das medidas obtidas durante a duração total do teste (6 minutos).

## Qualidade de vida relacionada à saúde

A QV relacionada à saúde foi realizada por entrevista utilizando o *Minnesota Living With Heart Failure Questionnaire* (MLWHFQ). O MLHFQ abrange 21 perguntas, cujo objetivo é determinar como a doença afetou as condições físicas, psicológicas e socioeconômicas dos pacientes no mês anterior.^20^ As perguntas incluem sintomas e sinais relevantes para a doença, níveis de atividade física, trabalho, interação social, atividade sexual e emoções. O escore total do MLHFQ varia de 0 a 105 (sem comprometimento a comprometimento máximo). Dois outros escores podem ser determinados: a dimensão física (8 itens, 0 a 40) e a dimensão emocional (5 itens, 0 a 25). Um escore maior no MLHFQ significa pior QV. As opções de respostas variam de 0 (nenhum) a 5 (muito), onde 0 representa nenhuma limitação e 105 representa limitação máxima.

## Análise estatística

A normalidade dos dados foi verificada pelo teste de Shapiro-Wilk. As variáveis não distribuídas normalmente foram transformadas em logaritmo natural (peso, massa gorda, massa livre de gordura, 8FUG, escore total de MLHFQ, MLHFQ físico e MLHFQ emocional) para análises subsequentes e então transformadas de volta à escala original para fins de clareza. Os dados são expressos como média ± desvio padrão. Os dados categóricos são apresentados como valores absolutos e porcentagens. A correlação de Pearson foi utilizada para analisar a relação entre os componentes da aptidão física (equilíbrio dinâmico e mobilidade, força da parte superior do corpo, ACR e IMC) para verificar a colinearidade entre as variáveis (r >0,75). A correlação parcial (ajustada por idade, sexo e classe da NYHA) foi utilizada para avaliar a associação entre os componentes da aptidão física e as dimensões da QV. Foi realizada análise de regressão linear multivariada, com seleção gradual de variáveis para determinar a relação entre as dimensões da QV com idade, sexo, classe funcional da NYHA e componentes da aptidão física, identificados como potenciais preditores independentes de QV. A análise estatística foi realizada utilizando o software IBM SPSS 24 (SPSS, EUA), e a significância estatística foi estabelecida em p <0,05.

## Resultados

### Característica dos pacientes

As características demográficas e clínicas dos pacientes são apresentadas na Tabela 1 . A média de idade dos pacientes foi de 76 ± 6 anos, variando de 59 a 85 anos, e 71% (n = 17) eram do sexo feminino. Hipertensão foi a comorbidade mais prevalente (n = 22, 92%), seguida por dislipidemia (n = 17, 71%) e obesidade (n = 14, 58%). Em relação à classe funcional da NYHA, 79% (n = 19) de todos os pacientes foram classificados na classe II. A média do BNP foi de 288,9 ± 191,5 pg/mL-1. Em relação à função cardíaca, a fração de ejeção média foi de 60 ± 6%, 23% (n = 6) dos pacientes apresentaram E / e’ >15, enquanto 90% (n = 22) apresentaram LAVI >34 mL/m^2^. Todos os pacientes apresentavam hipertrofia ventricular esquerda.

**Tabela 1:** – Características gerais dos pacientes

	Todas (n=24)
**Características Sociodemográficas**	
Idade (anos)	76 ± 6,1
Sexo feminino (n) (%)	17 (71%)
**Antropometria**	
Peso (Kg)	71,8 ± 15,9
Circunferência da cintura (cm)	100,9 ± 12,6
Gordura corporal (%)	36,1±6,5
Massa livre de gordura (kg)	45,4±9,7
**Risk factors, n (%)**	
Obesidade (IMC ≥ 30 kg/m2)	14 (58%)
Ex-fumante	4 (17%)
Hipertensão	22 (92%)
Dislipidemia	17 (71%)
Diabetes tipo 2	2 (8%)
Pré-diabético	9 (38%)
Fibrilação Atrial	12 (50%)
Fibrilação Atrial (paroxística)	4 (17%)
DPOC	2 (8%)
Apneia obstrutiva do sono	6 (25%)
**Sinais clínicos**	
FC de repouso (bpm)	72 ± 16
PAS (mmHg)	136 ± 19
PAD (mmHg)	70 ± 14
BNP (pg/mL)	289 ± 192
NYHA classe II	19 (79%)
NYHA classe III	4 (17%)
**Medicamentos (%)**	
IECA/BRA	17 (71%)
ß-Bloqueador	20 (83%)
Diuréticos de alça	18 (75%)
Estatina	16 (67%)
Digoxina	4 (17%)
ARM	2 (8%)
**Função Cardíaca**	
FEVE (%)	60 ± 6,3
E/e´	12,2 ± 3,1
E/A	1,0 ± 0,5
IMVE (gm/m2)	231,3 ± 94,5
IVAE (mL/m2)	44,2 ± 11,7

*DPOC: doença pulmonar obstrutiva crônica; FC: frequência cardíaca; PAS: pressão arterial sistólica; PAD: pressão arterial diastólica; BNP: peptídeo natriurético cerebral; NYHA: New York Heart Association; IECA/BRA: inibidor da enzima de conversão da angiotensina e bloqueador do receptor da angiotensina; ARM: antagonistas dos receptores mineralocorticoides; FEVE: fração de ejeção do ventrículo esquerdo; E/e’: razão entre velocidade inicial do fluxo mitral e velocidade diastólica inicial do anel mitral; E/A: razão onda diastólica de enchimento inicial/onda de enchimento tardio; IMVE: índice de massa do ventrículo esquerdo; IVAE: índice de volume do átrio esquerdo.*

### Qualidade de vida

O escore da escala total do MLHFQ foi de 26 ± 24, enquanto as subescalas física e emocional do MLHFQ foram de 12 ± 13 e 5 ± 7, respectivamente.

### Aptidão física

Em geral, a distância no TC6M, o 8FUG e os resultados do teste de preensão manual foram de 312 ± 90 metros 10,9 ± 3,6 segundos, e 18,6 ± 7,1 kg, respectivamente. O VO2 médio durante o teste foi de 11,2 ± 2,3 mL/ min-1 /kg-1. A correlação bivariada entre os componentes da aptidão física mostrou que o teste 8FUG estava inversamente correlacionado com o teste de preensão manual (r = -0,47; p = 0,01) e a distância no TC6M (r = -0,81; p> 0,001) ( Tabela 2 ).

**Tabela 2:** – Correlação bivariada entre parâmetros de aptidão física

	Equilíbrio dinâmico e mobilidade	Força na parte superior do corpo	Aptidão cardiorrespiratória	Composição corporal		

	8FUG	Preensão manual	TC6M	IMC	% MG	MLG
8FUG		-0,478 (0,018) (0,018)	-0,816 (<0,00)	-0,030 (0,888)	0,184 (0,389)	-0,221 (0,299)
Preensão manual	-0,478 (0,018)		0,390 (0,060)	0,017 (0,939)	-0,362 (0,082)	0,284 (0,179)
TC6M	-0,816 (<0,00)	0,390 (0,060)		-0,074 (0,733)	-0,161 (0,453)	0,010 (0,964)
IMC	-0,030 (0,888)	0,017 (0,939)	-0,074 (0,733)		0,566 (0,004)	0,533 (0,007)
MG	0,184 (0,389)	-0,362 (0,082)	-0,161 (0,453)	0,566 (0,004)		-0,258 (0,224)
MLG	-0,221 (0,299)	0,284 (0,179)	0,010 (0,964)	0,533 (0,007)	-0,258 (0,224)	

*8FUG: teste 8-foot up and go; TC6M: teste de caminhada de 6 minutos; IMC: índice de massa corporal; MG: massa gorda; MLG: massa livre de gordura. Dados são r (p).*

### Associação entre aptidão física e qualidade de vida

A correlação parcial entre as dimensões da QV e os componentes da aptidão física é apresentada na Tabela 3 . Um melhor escore total do MLHFQ foi diretamente correlacionado com o teste 8FUG (r = 0,563; p = 0,008) e inversamente correlacionado com o TC6M (r = -0,539; p = 0,012). Em relação ao MLHFQ físico, este foi diretamente correlacionado com os resultados do teste 8FUG (r = 0,529; p = 0,014) e inversamente correlacionado com o TC6M (r = -0,478 p = 0,028). Por fim, o MLHFQ emocional foi diretamente correlacionado com o teste 8FUG (r = 0,597; p = 0,004).

**Tabela 3:** – Correlação parcial entre dimensões da qualidade de vida com componentes da aptidão física

	Equilíbrio dinâmico e mobilidade	Força na parte superior do corpo	Aptidão cardiorrespiratória	Composição corporal		

	8FUG	Teste de preensão manual	TC6M	IMC	% MG	MLG
**MLHFQ total**	0,563 (0,008)	-0,118 (0,611)	-0,539 (0,012)	0,208 (0,366)	-0,012 (0,957)	0,372 (0,097)
**MLHFQ físico**	0,529 (0,014)	-0,261 (0,254)	-0,478 (0,028)	0,260 (0,255)	-0,027 (0,909)	0,353 (0,116)
**MLHFQ emocional**	0,597 (0,004)	-0,023 (0,919)	-0,394 (0,077)	0,199 (0,388)	0,002 (0,993)	0,297 (0,191)

*Ajustado por idade, sexo e classe funcional da NYHA. 8FUG: teste 8-foot up and go; TC6M: teste de caminhada de seis minutos. IMC: índice de massa corporal; MG: massa gorda; MLG: massa livre de gordura; MLHFQ: Minnesota Living with Heart Failure Questionnaire. Os dados são r (p).*

A tabela 4 mostra a análise de regressão multivariada para as dimensões da QV. Todos os modelos foram ajustados para idade, sexo e classe funcional da NHYA como potenciais fatores de confusão. Para o escore total do MLHFQ, o 8FUG foi o único parâmetro de aptidão física que permaneceu como preditor independente (β = 0,651; p = 0,001). Da mesma forma, para a dimensão física do MLHFQ, o 8FUG foi o único componente da aptidão física que permaneceu como preditor independente (β = 0,570; p = 0,04). Finalmente, para o MLHFQ emocional, o 8FUG foi o único componente da aptidão física que permaneceu como preditor independente (β = 0,611; p = 0,002).

**Tabela 4:** – Análise de regressão stepwise, avaliando quais componentes da aptidão física foram independentemente associados à dimensão específica da qualidade de vida

	β	B	R^2^	p
**MLHFQ total**				
Ln 8FUG	0,651	5,015	0,424	0,001
**MLHFQ físico**				
Ln 8FUG	0,570	3,788	0,324	0,040
**MLHFQ emocional**				
Ln 8FUG	0,611	3,003	0,373	0,002

*Ln 8FUG: logaritmo natural do teste 8-foot up and go; MLHFQ: Minnesota Living with Heart Failure Questionnaire; β: coeficiente de regressão padronizado; B: coeficiente de regressão não padronizado; R^2^: coeficiente de determinação ajustado.*

## Discussão

Os dados fornecidos pelo nosso estudo indicam que a aptidão física está positivamente correlacionada com a QV em pacientes com ICFEP. Além disso, o equilíbrio dinâmico e a mobilidade foram os únicos componentes da aptidão física independentemente associados ao escore total da QV e às dimensões física e emocional. Esses achados sugerem que esse componente específico da aptidão física supera a ACR na avaliação da QV dos pacientes com ICFEP. Além disso, destaca a necessidade de estudar intervenções visando especificamente esses componentes de condicionamento físico para aumentar os ganhos de QV.

Apesar da alta prevalência e do prognóstico ruim da ICFEP, terapias baseadas em evidências ainda precisam ser desenvolvidas para reduzir de forma efetiva a morbidade ou a mortalidade.^4 , 5^ Esses pacientes geralmente são caracterizados por baixa QV21 e as diretrizes atuais de tratamento destacam a importância de se melhorar o bem-estar dos pacientes.^5^ A aptidão física é um constructo com múltiplos componentes^8^ e vários estudos demonstram que é um dos principais determinantes da QV na ICFEP.^6 , 7^ Nossos resultados corroboram esse achado, uma vez que observamos que o escore total da QV correlacionava-se fortemente com a aptidão física (por exemplo, equilíbrio dinâmico e mobilidade e ACR) em pacientes com ICFEP.

Como a aptidão física pode influenciar a QV, estratégias direcionadas à aptidão física podem potencialmente melhorar a QV, independentemente de outros benefícios à saúde.^22^ Uma metanálise recente mostrou que a combinação de treinamento físico de resistência com medicamentos cardiovasculares fornece uma melhora clinicamente relevante na capacidade de exercício e QV em pacientes com ICFEP.^23^ Entretanto, a aptidão física e a QV são constructos multicomponentes e multidimensionais, respectivamente, e é crucial determinar qual dimensão/componente está melhor relacionado entre si para maximizar possíveis melhorias na QV.

Estudos anteriores demonstraram que a ACR está principalmente associada à dimensão física, mas não necessariamente ao escore total ou dimensão emocional da QV.^7 , 10^ Observamos que a ACR (avaliada pelo TC6M) e o equilíbrio dinâmico e mobilidade (avaliado pelo teste 8FUG) foram ambos associados às dimensões físicas da QV. Além disso, o equilíbrio dinâmico e a mobilidade foram o único componente da aptidão física associado à dimensão emocional da QV, enquanto a força da parte superior do corpo (avaliada pelo Teste de força de preensão manual) e a composição corporal não foram associadas a nenhuma dimensão. Além disso, análises multivariadas mostraram que o equilíbrio dinâmico e a mobilidade foram o único componente da aptidão física independentemente associado a todas as dimensões da QV, explicando 42% de variação no escore total da QV, 32% da dimensão física e 37% da dimensão emocional da QV. Assim, de todos os componentes da aptidão física, o equilíbrio dinâmico e a mobilidade parecem ser os que melhor avaliam a QV em pacientes com ICFEP.

Coletivamente, nossos dados sugerem que a melhora do componente específico da aptidão física do equilíbrio dinâmico e da mobilidade proporcionará, eventualmente, uma maior melhora na QV. O teste 8FUG reflete as demandas específicas de atividades como levantar-se de uma posição sentada, caminhar pequenas distâncias, voltar, parar e sentar-se.^24^ Isso pode ser explicado pela ampla gama de habilidades físicas, incluindo menor força corporal, equilíbrio dinâmico, capacidade de caminhar, agilidade e velocidade da marcha8 envolvidas no teste 8FUG. Essas habilidades também são necessárias nas tarefas diárias normais de uma vida independente e autônoma, especialmente em idosos.^25^ Estudos futuros (por exemplo, programas de treinamento longitudinal) devem avaliar se um programa de treinamento físico concentrado no aprimoramento das habilidades motoras (por exemplo, equilíbrio dinâmico e mobilidade) pode melhorar os componentes físicos e emocionais da QV na ICFEP em comparação com os padrões atuais.

### Limitações do estudo

O pequeno tamanho da amostra, o desenho transversal e de conveniência de nosso estudo limitam a generalização de nossos resultados. Apesar disso, nossa amostra reúne as características clínicas usuais da população com ICFEP relatadas em grandes estudos,^5^ com maior prevalência de mulheres idosas e maior prevalência de comorbidades. Mais estudos prospectivos de coorte com um tamanho amostral maior são necessários para fortalecer ou refutar nossas conclusões de que o equilíbrio dinâmico e a mobilidade são mais eficientes para avaliar a QV dos pacientes com ICFEP.

## Conclusão

No geral, nossos achados indicam que tanto a ACR quanto o equilíbrio dinâmico e a mobilidade estão diretamente associados ao escore total e às dimensões físicas da QV em pacientes com ICFEP, mas apenas o equilíbrio dinâmico e a mobilidade foram associados concomitantemente à dimensão emocional. Análises multivariadas revelaram que o equilíbrio dinâmico e a mobilidade superam a ACR na análise da QV de pacientes com ICFEP. Além disso, nossos dados sugerem que direcionar o tratamento especificamente para a agilidade e o equilíbrio motor pode ser uma estratégia importante para aprimorar os ganhos de QV em todas as dimensões.
