# Supplementary material for: Equilíbrio Dinâmico e Mobilidade Explicam a Qualidade de Vida na ICFEP, Superando Todos os Outros Componentes da Aptidão Física
Source: Arq Bras Cardiol. 2020 May 12;114(4):701–7. [Article in Portuguese] doi: 10.36660/abc.20190080 (PMC9744334; doi:10.36660/abc.20190080)
Supplement: Translation

# Translation: Dynamic Balance and Mobility Explain Quality of Life in HFpEF, Outperforming All the Other Physical Fitness Components

**Authors:** Cristine Schmidt, Mário Santos, Lucimere Bohn, Bruno Miguel Delgado, Daniel Moreira-Gonçalves, Adelino Leite-Moreira, José Oliveira

**Affiliations:** 1 Portugal Faculdade de Desporto da Universidade do Porto (FADEUP), Centro de Investigação em Actividade Física, Saúde e Lazer (CIAFEL), Porto – Portugal; 2 Portugal Instituto de Ciências Biomédicas Abel Salazar (ICBAS), Universidade do Porto, Unidade de Pesquisa Multidisciplinar em Biomedicina, Porto – Portugal; 3 Portugal Centro Hospitalar do Porto, Departamento de Cardiologia, Porto – Portugal; 4 Portugal Centro Hospitalar do Porto, Porto – Portugal; 5 Portugal Faculdade de Medicina da Universidade do Porto (FMUP), Unidade de Investigação Cardiovascular (UniC), Porto – Portugal

**Keywords:** Heart Failure/physiopathology, Work-Life Balance/methods, Physical Fitness, Quality of Life, Breathing Exercises, Body Composition

## Abstract

**Background:**

Physical fitness is an important determinant of quality of life (QoL) in heart failure with preserved ejection fraction (HFpEF) patients. However, how the different physical fitness components correlate with the specific dimensions of QoL in HFpEF patients remains unknown.

**Objective:**

To evaluate the association between different physical fitness components and QoL dimensions in HFpEF patients, and, assess which physical fitness components were independently associated to QoL.

**Methods:**

Patients with HFpEF were assessed for physical fitness [dynamic balance and mobility (8-foot-up-and go test), upper body strength (handgrip), cardiorespiratory fitness (CRF) (6-minute-walking test) and body composition (body mass index)] and for QoL (Minnesota Living With Heart Failure Questionnaire). Partial correlation was used to verify the association between physical fitness components and QoL dimensions. The determination of independent predictors in QoL dimensions was assessed through stepwise multivariate linear regression analysis. Statistical significance was set at p<0.05.

**Results:**

Both CRF and dynamic balance and mobility are significantly associated with the total score and physical dimensions of QoL (p<0.05), but only dynamic balance and mobility were concomitantly associated with the emotional dimension (r=0.597; p=0.004). Dynamic balance and mobility were independently associated with total score (β=0.651; r^2^=0.424; p=0.001), physical (β=0.570; r^2^=0.324; p=0.04) and emotional (β=0.611; r2=0.373 p=0.002) dimensions of QoL.

**Conclusion:**

Our data suggests that dynamic balance and mobility better assess QoL than CRF, which is commonly measured in clinical practice. Whether interventions specifically targeting dynamic balance and mobility have different impacts on QoL remains unknown. (Arq Bras Cardiol. 2020; 114(4):701-707)

## Introduction

Heart failure with preserved ejection fraction (HFpEF) accounts for half of all HF cases in the developed world’s population.^1^ The most common manifestation of the disease is exercise intolerance, which impacts on patients’ ability to cope with activities of daily life and reduces their quality of life (QoL).^2^ Furthermore, QoL is related with poor outcomes, such as higher frequency of hospital readmission and higher mortality rates.^3^ Despite its high prevalence and poor prognosis, HFpEF remains a disease with no approved therapy that improves survival.^4^ Therefore, current recommendations for the treatment of these patients highlight the importance to focus on effective therapies capable of alleviating symptoms and meaningfully improve QoL.^5^

Reduced levels of physical fitness are associated with poor QoL in patients with HFpEF.^6 , 7^ Importantly, exercise training has shown to improve physical fitness, together with symptom and QoL improvement.^6 , 7^ Because physical fitness and QoL are mutually related, targeting physical fitness with exercise training programs may be an effective strategy to accomplish the management recommendations of patients with HFpEF.^5^ However, physical fitness is a multicomponent (e.g. dynamic balance and mobility, muscular fitness, cardiorespiratory fitness (CRF) and body composition)^8^ and, in parallel, QoL is a multidimensional construct (e.g. general, emotional and physical).^9^ Until now, it remains unknown how the different components of physical fitness correlate with the specific dimensions of QoL in HFpEF patients. To date, it has only been demonstrated that a higher CRF is associated with a better QoL, mainly regarding the physical dimension.^7 , 10^ However, the influence of other physical fitness components on QoL dimensions is relatively unknown. Therefore, the clarification of this issue might have important clinical implications in the design of specific interventional programs for HFpEF patients targeting the physical fitness component, which most impacts on QoL, whether overall or one of its depressed dimensions.

Therefore, the aims of the present study are twofold: i) to evaluate the association between different physical fitness components (CRP, upper body strength, dynamic balance and mobility, and body composition) and the QoL dimensions (total, physical and emotional) in HFpEF patients, and ii) to assess which of the physical fitness components are independently associated to different dimensions of QoL in this specific population.

## Methods

### Study design

This is a cross-sectional study conducted in a Portuguese public hospital ( *Centro Hospitalar do Porto - Hospital de Santo Antonio* , Porto) with a convenience sample of HFpEF. Inclusion criteria was diagnosis of HFpEF according to the European Society of Cardiology guidelines.^11^ Patients were excluded if they presented with unstable angina, acute coronary syndrome as primary diagnosis, symptomatic severe aortic stenosis, acute pulmonary embolism, acute myocarditis, decompensated heart failure, uncontrolled hypertension, complex ventricular arrhythmias, severe renal dysfunction, severe chronic obstructive pulmonary disease, medical or orthopedic conditions that precluded independent ambulation and exercise testing.

Patients who were potentially eligible to participate in the study were identified from the clinical files of the hospital cardiology department. A total of 30 patients were invited through phone calls by a cardiologist. Of those, 24 patients (17 women and 7 men) accepted to take part in the study. The study was approved by the Ethics Committee of *Centro Hospitalar do Porto - Hospital de Santo Antonio* ( *N/S:* 2015.125) and met the ethical standards of the Declaration of Helsinki.

Data were collected from November 2016 to September 2017 during a single day in the hospital.

### Data collection

#### Blood pressure

A trained researcher performed blood pressure measurements after a 10 minute-rest in the sitting position. Blood pressure was assessed (Colin, BP 8 800; Critikron, Inc., USA) in both arms, and the arm showing the highest BP was used. SBP and DBP were computed as the average of 3 readings, with a 2-minute interval between them. Additional readings were performed when differences between readings exceeded 5 mmHg.^12^

## Blood collection and biochemical determinations

Peripheral venous blood (15 mL) was collected into an EDTA tube. The EDTA tubes were immediately placed on ice and allowed to clot for 30 minutes before centrifugation for 15 minutes at 1000xg. The plasma was aliquoted and stored at -80ºC for biochemical analysis. Brain Natriuretic Peptide (BNP) was quantified in a certified laboratory using chemiluminescent microparticle immunoassay (ARCHITECT BNP).

## Anthropometric and body composition measures

Body height (cm) was measured in the upright position using a stadiometer (Holtain Ltd., Crymmych, UK).^13^ Weight (kg), body mass index (BMI; kg·m^2^), fat mass (%) and free fat mass (kg) were measured with patients wearing light clothes, using an electronic segmental body composition analyzer (Tanita, BC-418, Tokyo, Japan). Fat mass and free fat mass were measured using bioelectrical impedance. Patients were asked to fast for 10-12 hours, avoid vigorous physical exercise and alcohol intake before being measured. Waist circumference (cm) was measured at the midpoint between the lowest rib and the iliac crest at the end of normal expiration.^14^ Obesity was determined as BMI equal or higher than 30 kg/m^2^ .^15^

## Functional classification

Patients were classified by the physician into subgroups based on their symptoms using the New York Heart Association (NYHA) functional class. Patients’ symptoms are based on how much they were limited during physical activity (class I to IV).^16^

## Echocardiography Evaluation

Supine transthoracic echocardiography was performed using a cardiovascular ultrasound model Vivid E95^®^(GE Healthcare). All quantitative echocardiographic measurements were performed by a single reader blinded to the results of the other evaluations, using a computerized off-line analysis station. Peak early diastolic tissue velocity was measured at the septal and lateral mitral annulus. Mitral inflow velocity was assessed by pulsed wave Doppler at the apical 4-chamber view, positioning the sample volume at the tip of the mitral leaflets. E/e’ ratio was calculated as E wave divided by e’ velocities. LV mass was estimated based on LV linear dimensions and indexed to body surface area, as recommended by ESC guidelines.17 LV hypertrophy was defined as LV mass indexed to body surface area (LV mass index) >115 g·m^2^in men or >95 g·m^2^in women. LV volumes were estimated by the modified Simpson method using the apical 4- and 2-chamber views, and LVEF was derived from volumes in the standard manner. LA volume was estimated by the method of disks using apical 4- and 2-chamber views at an end-systolic frame preceding mitral valve opening and was indexed to body surface area to derive LA volume index.

## Physical Fitness

**Dynamic balance and mobility.** It was assessed with the 8-foot up and go (8FUG) test.^18^ The patient starts the evaluation in the sitting position. After a signal, the patient must stand up, walk 8 feet (2.44m), make a turn around a cone, and return back to the initial position as fast as possible.^18^ The patients tried to perform the test twice. Time (in seconds) to complete each trial was measured with a stopwatch and the result considered was the shorter time.^19^

**Upper body strength.** Grip strength (kg) was isometrically measured using a Lafayette Instrument Hand dynamometer ( *Model 78010, 78011, Indiana, USA* ). Both arms were measured 3 times while patients were seated, with shoulder adducted and neutrally rotated, the elbow flexed at 90°, and the forearm and wrist in a neutral position. The average between attempts was used as final score for each arm.^19^

**Cardiorespiratory fitness with pulmonary gas exchange assessment.** It was assessed by the 6-minute walk test (6MWT) in a 25-m-long unobstructed corridor. Participants were instructed to walk the maximal distance in 6 minutes time. Resting stops were allowed when patients felt it to be necessary. The 6MWT was performed wearing a portable gas analyzer (K4b2, Cosmed, Rome, Italy) and a heart rate monitor (Polar Electro Oy, Kempele, Finland). Oxygen uptake (VO2; mL·min-1·kg-1) and heart rate (HR; bpm) were measured directly and continuously. Respiratory and HR measurements were collected in a breath-by-breath and beat-to-beat basis, respectively, and then, data were averaged over 5-s intervals. Data was calculated as the average of measures taken during the test total duration (6 minutes).

## Health-related quality of life

Health-related QoL was measured through an interview using the Minnesota Living With Heart Failure Questionnaire (MLWHFQ). The MLHFQ encompasses 21 questions, whose purpose is to determine how disease affects the physical, psychological and socioeconomic conditions of the patients during the previous month.^20^ The questions include symptoms and signs relevant to disease, levels of physical activity, work, social interaction, sexual activity, and emotions. The MLHFQ total score range from 0 to 105 (no impairment to maximum impairment). Two other scores can be determined: the physical dimension (8 items, 0–40), and the emotional dimension (5 items, 0–25). A higher MLHFQ score means a worse QoL. Answers options ranges from 0 (none) to 5 (very much), where 0 represents no limitation and 105 represents maximal limitation.

## Statistical analyses

Data normality was verified by Shapiro-Wilk test. Non-normally distributed variables were transformed into a natural logarithm (weight, fat mass, free fat mass, 8FUG, MLHFQ total score, MLHFQ physical and MLHFQ emotional) for subsequent analysis and then transformed back to the original scale for the purpose of clarity. Data are expressed as mean ± standard deviation. Categorical data are reported as absolute values and percentages. Pearson’s correlation was used to analyze the association between physical fitness components (dynamic balance and mobility, upper body strength, CRF and BMI) aiming to verify collinearity between variables (r>0.75). Partial correlation (adjusted for age, gender and NYHA class) was used to assess the association between physical fitness components and QoL dimensions. A multivariate linear regression analysis, with stepwise selection of variables, was performed to determine the association between QoL dimensions and age, gender, NYHA functional class and physical fitness components, which were identified as potential independent predictors of QoL. The statistical analysis was performed using the IBM SPSS 24 software (SPSS, USA), and the statistical significance was set at p<0.05.

## Results

### Patients’ characteristics

The demographic and clinical characteristics of patients are shown in Table 1 . The patients’ mean age was 76±6 years old, ranging from 59 to 85 years, and 71% (n=17) were females. Hypertension was the most prevalent comorbidity (n=22, 92%), followed by dyslipidemia (n=17, 71%) and obesity (n=14, 58%). Regarding the NYHA functional class, 79% (n=19) of all patients were classified as class II. The average BNP level was 288.9±191.5 pg·mL-1. Regarding cardiac function, the mean ejection fraction was 60±6%, 23% (n=6) of patients had E/e` >15, while 90% (n=22) had LAVI >34 mL·m^2^. All patients had left ventricular hypertrophy.

**Table 1:** – General patients’ characteristics

	All (n=24)
**Sociodemographic characteristics**	
Age (years)	76 ± 6.1
Female (n) (%)	17 (71%)
**Anthropometrics**	
Weight (Kg)	71.8 ± 15.9
Waist circumference (cm)	100.9 ± 12.6
Body fat (%)	36.1±6.5
Free fat mass (kg)	45.4±9.7
**Risk factors, n (%)**	
Obesity (BMI ≥ 30 kg/m2)	14 (58%)
Ex-smoker	4 (17%)
Hypertension	22 (92%)
Dyslipidemia	17 (71%)
Type 2 diabetes	2 (8%)
Pre-diabetic	9 (38%)
Atrial fibrillation	12 (50%)
Atrial fibrillation (paroxysmal)	4 (17%)
COPD	2 (8%)
Obstructive sleep apnea	6 (25%)
**Clinical signs**	
Resting HR (bpm)	72 ± 16
SBP (mmHg)	136 ± 19
DBP (mmHg)	70 ± 14
BNP (pg/mL)	289 ± 192
NYHA class II	19 (79%)
NYHA class III	4 (17%)
**Medication (%)**	
ACE-i/ARB	17 (71%)
ß-Blocker	20 (83%)
Loop diuretics	18 (75%)
Statin	16 (67%)
Digoxin	4 (17%)
MRAs	2 (8%)
**Cardiac Function**	
LVEF (%)	60 ± 6.3
E/e´	12.2 ± 3.1
E/A	1.0 ± 0.5
LVMI (gm/m^2^)	231.3 ± 94.5
LAVI (mL/m^2^)	44.2 ± 11.7

*COPD: chronic obstructive pulmonary disease; HR: heart rate; SBP: systolic blood pressure; DBP: diastolic blood pressure; BNP: brain natriuretic peptide; NYHA: New York Heart Association; ACEi/ARB: angiotensin-converting enzyme inhibitor and angiotensin receptor blocker; MRAs: mineralocorticoid receptor antagonists; LVEF: left ventricle ejection fraction; E/e´: ratio of early mitral inflow velocity and mitral annular early diastolic velocity; E/A: mitral ratio of peak early to late diastolic filing velocity; LVMI: left ventricle mass index; LAVI: left atrium volume index.*

### Quality of life

The score of total MLHFQ scale was 26±24, whereas the physical and emotional MLHFQ subscales’ scores were 12±13, and 5±7, respectively.

### Physical fitness

Overall, the 6MWT distance, 8FUG and handgrip results were 312±90 meters, 10.9±3.6 seconds, and 18.6±7.1kg, respectively. The mean VO_2_during the test was 11.2± 2.3 mL·min-1·kg-1. A bivariate correlation between physical fitness components showed that the 8FUG test was inversely correlated with handgrip (r=-0.47; p=0.01) and 6MWT distance (r=-0.81; p>0.001) ( Table 2 ).

**Table 2:** – Bivariate correlation between physical fitness parameters

	Dynamic balance and mobility	Upper body strength	Cardiorespiratory fitness	Body composition		

	8FUG	Handgrip	6MWT	BMI	% FM	FFM
8FUG		-0.478 (0.018)	-0.816 (<0.00)	-0.030 (0.888)	0.184 (0.389)	-0.221 (0.299)
Handgrip	-0.478 (0.018)		0.390 (0.060)	0.017 (0.939)	-0.362 (0.082)	0.284 (0.179)
6MWT	-0.816 (<0.00)	0.390 (0.060)		-0.074 (0.733)	-0.161 (0.453)	0.010 (0.964)
BMI	-0.030 (0.888)	0.017 (0.939)	-0.074 (0.733)		0.566 (0.004)	0.533 (0.007)
Fat Mass	0.184 (0.389)	-0.362 (0.082)	-0.161 (0.453)	0.566 (0.004)		-0.258 (0.224)
FFM	-0.221 (0.299)	0.284 (0.179)	0.010 (0.964)	0.533 (0.007)	-0.258 (0.224)	

*8FUG: 8-foot up and go test; 6MWT: six-minute walk test; BMI: body mass index; FM: fat mass; FFM: free fat mass. Data are r (p).*

### Association between physical fitness and quality of life

A partial correlation between QoL dimensions and physical fitness components are shown in Table 3 . A better MLHFQ total score was directly correlated with 8FUG (r=0.563; p=0.008) and inversely correlated with 6MWT (r=-0.539; p=0.012) test results. Regarding MLHFQ physical, it was directly correlated with 8FUG (r=0.529; p=0.014) and inversely correlated with 6MWT (r=-0.478 p=0.028). Finally, MLHFQ emotional was directly correlated with 8FUG (r=0.597; p=0.004).

**Table 3:** – Partial correlation between quality of life dimensions and physical fitness components

	Dynamic balance and mobility	Upper body strength	Cardiorespiratory fitness	Body composition		

	8FUG	Handgrip	6MWT	BMI	% FM	FFM
**MLHFQ total**	0.563 (0.008)	-0.118 (0.611)	-0.539 (0.012)	0.208 (0.366)	-0.012 (0.957)	0.372 (0.097)
**MLHFQ physical**	0.529 (0.014)	-0.261 (0.254)	-0.478 (0.028)	0.260 (0.255)	-0.027 (0.909)	0.353 (0.116)
**MLHFQ emotional**	0.597 (0.004)	-0.023 (0.919)	-0.394 (0.077)	0.199 (0.388)	0.002 (0.993)	0.297 (0.191)

*Adjusted for age, gender and NYHA functional class. 8FUG, 8-foot up and go test. 6MWT, six-minute walk test. BMI, body mass index. FM, fat mass. FFM, free fat mass. MLHFQ, Minnesota Living with Heart Failure Questionnaire. Data are r (p).*

Table 4 shows the multivariate regression analysis for QoL dimensions. All models were adjusted for age, gender and NHYA functional class as potential confounders. For MLHFQ total score, the 8FUG was the only physical fitness parameter that remained an independent predictor (β=0.651; p=0.001). Similarly, for MLHFQ physical dimension, the 8FUG was the single physical fitness component that remained an independent predictor (β=0.570; p=0.04). Finally, for MLHFQ emotional, the 8FUG was the single physical fitness component that remained an independent predictor (β=0.611; p=0.002).

**Table 4:** – Stepwise regression analysis assessing which physical fitness components were independently associated with specific quality of life dimensions

	β	B	R2	p
**MLHFQ total**				
Ln 8FUG	0.651	5.015	0.424	0.001
**MLHFQ physical**				
Ln 8FUG	0.570	3.788	0.324	0.040
**MLHFQ emotional**				
Ln 8FUG	0.611	3.003	0.373	0.002

*Ln 8FUG: natural logarithm of 8-foot up and go test; MLHFQ: Minnesota Living with Heart Failure Questionnaire; β: standardized regression coefficient; B: non-standardized regression coefficient; R^2^: adjusted coefficient of determination.*

## Discussion

The data provided by our study indicates that physical fitness is positively correlated with QoL in HFpEF patients. In addition, dynamic balance and mobility was the only physical fitness component that was independently associated with QoL total score, and physical and emotional dimensions. These findings suggest that this specific component of physical fitness outperforms CRF in assessing HFpEF patients’ QoL. In addition, it highlights the need to study interventions specifically targeting these fitness components to enhance QoL gains.

Despite the high prevalence and poor prognosis of HFpEF, evidence-based therapies aimed at effectively reducing morbidity or mortality remains to be developed.^4^ These patients are often characterized by poor QoL^21^ and current treatment guidelines highlight the importance of aiming to improve patients’ well-being.^5^ Physical fitness is a multicomponent construct^8^ and several studies show it is a major determinant of QoL in HFpEF.^6 , 7^ Our results corroborate this finding, as we observed that QoL total score strongly correlated with physical fitness (e.g. dynamic balance and mobility, and CRF) in HFpEF patients.

Because physical fitness might influence QoL, strategies targeting physical fitness might potentially improve QoL, independent of further health benefits.^22^ A recent meta-analysis showed that the combination of endurance exercise training together with cardiovascular drugs provide a clinically relevant improvement in both exercise capacity and QoL in HFpEF patients.^23^ However, physical fitness and QoL are multicomponent and multidimensional constructs, respectively, and it is crucial to ascertain which dimension/component is better related to each other to maximize possible QoL improvements.

Previous studies have shown that CRF is mainly associated with the physical dimension, but not necessarily with the total score or emotional dimension of QoL.^7 , 10^ We observed that CRF (assessed by the 6MWT) and dynamic balance and mobility (assessed by 8FUG) were both associated with the physical dimensions of QoL. Moreover, dynamic balance and mobility were the only physical fitness components associated with the QoL emotional dimension, while upper body strength (assessed by handgrip) and body composition were not associated with any dimension. In addition, multivariate analysis revealed that the dynamic balance and mobility was the only physical fitness component independently associated with all QoL dimensions, explaining 42% of variance in the total score QoL, 32% of the physical dimension and 37% of the emotional dimension of QoL. Thus, of all physical fitness components, dynamic balance and mobility seems to be the one that better assess QoL in HFpEF patients.

Collectively, our data suggest that improving the specific physical fitness component of dynamic balance and mobility will eventually result in the greatest QoL improvement. The 8FUG reflects the specific demands of activities, such as standing up from a sitting position, walking short distances, turning, stopping and sitting down.^24^ This might be explained by the wide range of physical abilities, including lower body strength, dynamic balance, walking ability, agility and gait speed^8^ involved in the 8FUG. These abilities are also required during the normal daily tasks of an independent and autonomous life, especially among the elderly.^25^ Future studies (e.g. longitudinal training programs) should assess if an exercise training program focused on enhancing motor abilities (e.g. dynamic balance and mobility) can improve the physical and emotional components of QoL in HFpEF in comparison to current standard ones.

### Study limitations

The small sample size, cross-sectional and convenience sampling design of our study limits the generalization of our results. Despite that, our sample assembles the usual clinical features of HFpEF population reported in large studies^5^ with a higher prevalence of elderly women and a higher prevalence of comorbidities. Further prospective cohort studies with a larger sample size are needed to strengthen or refute our conclusions that dynamic balance and mobility are more efficient in assessing HFpEF patients’ QoL.

## Conclusion

Overall, our findings indicate that both CRF and dynamic balance and mobility are directly associated with the QoL total score and physical dimensions in patients with HFpEF, but only dynamic balance and mobility were concomitantly associated with the emotional dimension. Multivariate analyses revealed that dynamic balance and mobility outperforms CRF in assessing HFpEF patients’ QoL. In addition, our data suggests that specifically targeting motor agility and balance may be an important strategy to enhance QoL gains in all dimensions.
